# Age, preoperative tumor volume and widening of the internal acoustic meatus are independent factors associated with poor preoperative hearing in vestibular schwannoma patients – results of a single-center retrospective analysis

**DOI:** 10.1007/s10143-024-02419-8

**Published:** 2024-06-08

**Authors:** Lorenz Dörner, Elisa-Maria Suhm, Vanessa Ries, Vitor Goncalves, Marco Skardelly, Jens Schittenhelm, Marcos Tatagiba, Felix Behling

**Affiliations:** 1 Department of Neurosurgery, University Hospital Tübingen, Eberhard-Karls-University Tübingen, Hoppe-Seyler Street 3, Tübingen, Germany; 2 Center for Neuro-Oncology, Comprehensive Cancer Center Tübingen-Stuttgart, University Hospital Tübingen, Eberhard-Karls-University Tübingen, Tübingen, Germany; 3 Faculty of Medicine, University of Porto, Porto, Portugal; 4 Department of Neurosurgery, Municipal Hospital Reutlingen, Reutlingen, Germany; 5 Department of Neuropathology, University Hospital Tübingen, Eberhard-Karls-University Tübingen, Tübingen, Germany; 6 Hertie Institute for Clinical Brain Research, Tübingen, Germany

**Keywords:** Vestibular schwannoma, Acoustic neurinoma, Preoperative hearing, Non-serviceable hearing, IAC, Internal acoustic canal

## Abstract

Preoperative hearing function shows wide variations among patients diagnosed with vestibular schwannoma. Besides the preoperative tumor size there are other factors that influence the preoperative hearing function that are frequently discussed. A comprehensive analysis of a large cohort of vestibular schwannomas has the potential to describe new insights and influence the preoperative management. We analyzed clinical factors, imaging data and the expression of the proliferation marker MIB1 as potential influencing factors on the preoperative hearing function in a retrospective cohort of 523 primary sporadic vestibular schwannomas. The results of the preoperative audiometry were quantified using the Gardner-Robertson Score. Uni- and multivariate analyses were performed. Serviceable hearing (Gardner-Robertson class 1 or 2) was documented in 391 patients (74.8%). Factors associated with non-serviceable hearing (Gardner-Robertson class 3–5) were patients of older age (*p* < 0.0001), larger preoperative tumor volume (*p* = 0.0013) and widening of the internal acoustic meatus compared to the healthy side (*p* = 0.0353). Gender and differences in the expression of the proliferation marker MIB1 had no influence on preoperative hearing. In the multivariate nominal logistic regression older age (OR 27.60 (CI 9.17–87.18), *p* < 0.0001), larger preoperative tumor volume (OR 20.20 (CI 3.43–128.58), *p* = 0.0011) and widening of the internal acoustic canal (OR 7.86 (CI 1.77–35.46), *p* = 0.0079) remained independent factors associated with non-serviceable hearing. Widening of the internal acoustic canal is an independent factor for non-serviceable preoperative hearing in vestibular schwannoma patients together with older age and larger preoperative tumor volume.

## Introduction

Vestibular schwannoma (VS) is the most common tumor located in the cerebellopontine angle (CPA), deriving from Schwann cells that form the myelin of the eighth cranial nerve, the vestibulocochlear nerve. The histopathology of this slow growing tumor is of benign nature [1]. VS has an incidence of 1.52 new cases per 100,000 inhabitants per year [2]. Patients typically present with unilateral sensorineural hearing impairment, as well as tinnitus, imbalance and vertigo. In large tumors additional disturbance of the facial or trigeminal nerve can be observed [3, 4]. Treatment strategies consist of watchful waiting, radiation therapy and microsurgical resection, depending on the clinical presentation, tumor extension as well as patient preferences [5].

Pure-tone average (PTA) and acoustic evoked potentials (AEP) are established strategies for assessing hearing function in VS patients [6]. Several factors are influential on the preoperative hearing in VS, with the most obvious parameter being preoperative tumor size. It is widely accepted that large initial tumor extension and tumor growth negatively affect the preoperative hearing function in VS patients [7, 8]. Pressure-induced nerve damage as well as compromise of the vascular supply by the labyrinthine artery are established pathophysiological mechanisms causing sensorineural hearing impairment in VS [9, 10].

However, variabilities in hearing function can be observed regardless of tumor size. Patients with small tumors may present with poor preoperative hearing at diagnosis, and on the other hand, quite extensive schwannoma size can be found in patients with little or no sensorineural hearing impairment. Therefore, the sole size of a schwannoma does not seem to adequately explain the mechanism of sensorineural hearing impairment [11]. Other influencing factors apart from tumor extension have been increasingly discussed, especially tumor-specific immune-related mechanisms [11–13] that illuminate new aspects of the interaction of tumor cells and the surrounding structures.

**Fig. 1:**
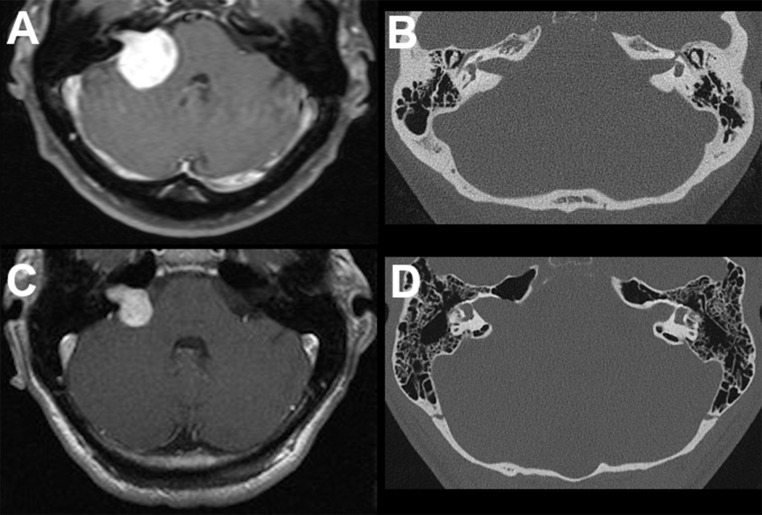
Two examples of vestibular schwannomas show that tumor size does not always correspond with widening of the internal acoustic canal. Large tumor extension (**A**) can be without widening of the IAC (**B**), while smaller tumor size (**C**) may be associated with marked IAC widening (**D**)

Berrettini et al. showed, that VS located in the internal acoustic canal seem to cause early hearing loss compared to VS growing in the CPA with the largest proportion in the cerebellopontine cistern [14]. Moreover, it is known, that a greater intracanalicular extent of VS is connected to heightened pressure in the IAC and is furthermore associated with preoperative hearing impairment [15, 16]. Widening of the IAC can be seen on preoperative computed tomographies [17] and may be a portrayal of the increased pressure in this area. Varieties of IAC widening independent of tumor size are quite common, as shown by two examples in Fig. 1.

To our knowledge, the first and only description of IAC abnormalities and hearing function in schwannomas dates back to 1997 [18]. However, a comprehensive multivariate approach in a large cohort of VS, including other established factors that influence hearing, has not yet been conducted to elucidate if IAC widening can be used as a reliable marker for preoperative hearing function.

## Materials and methods

### Patient cohort and clinical data

**Fig. 2:**
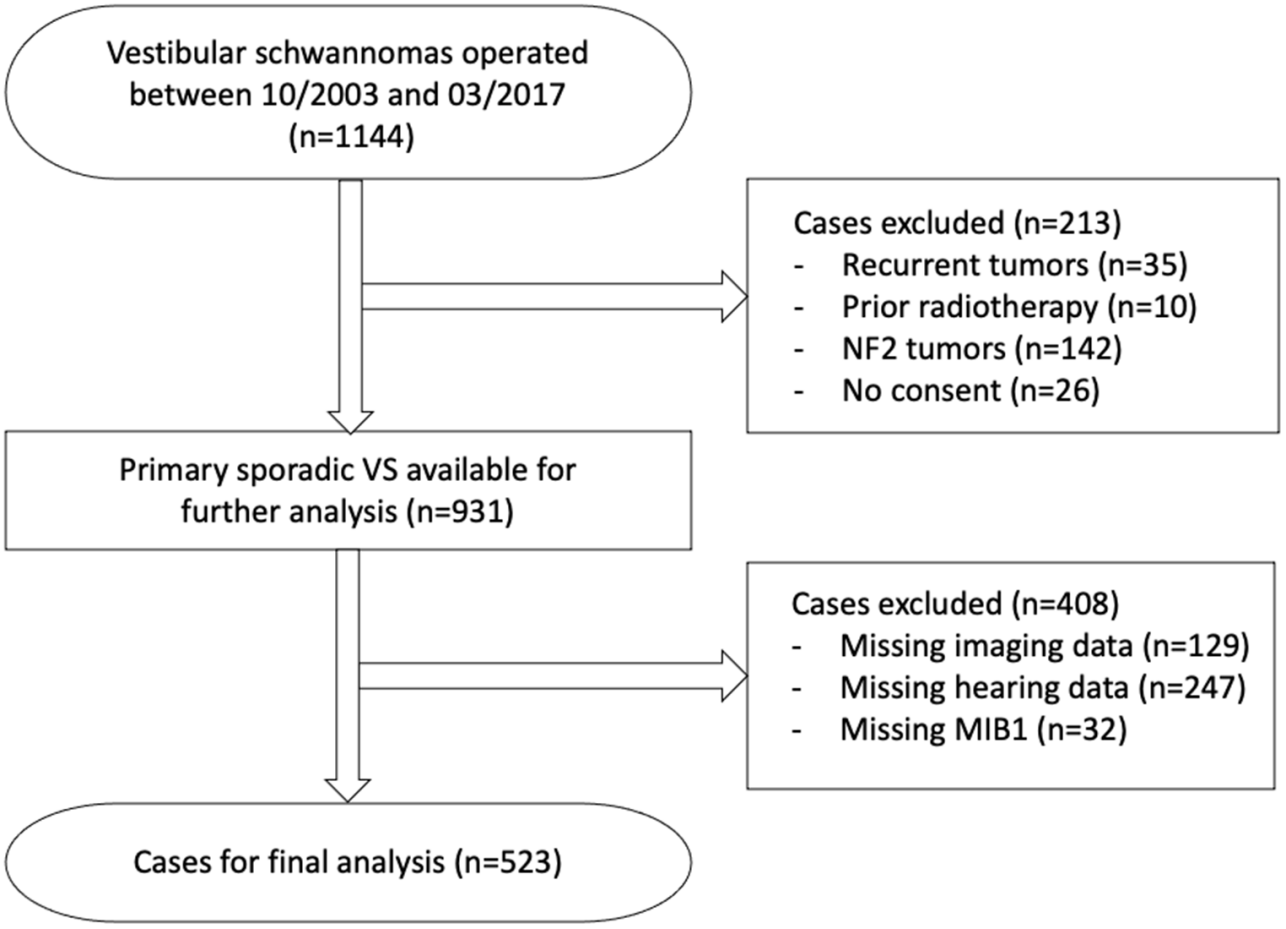
Flow chart delineating all excluded cases and the composition of the study cohort

We performed a retrospective analysis of all patients that were surgically treated in our department between October 2003 and March 2017 (*n* = 1144). Clinical data was collected by reviewing electronic patient data. Age at diagnosis, gender, prior treatments (surgery or radiation), presence of neurofibromatosis type 2 and preoperative hearing (pure tone audiogram) were collected. The following cases were excluded: NF2 (*n* = 142), recurrent tumors (*n* = 45), missing consent (*n* = 26), missing MIB1 data (*n* = 32), missing preoperative imaging (*n* = 129) and missing preoperative pure tone audiogram (*n* = 247). Complete datasets were available for 523 primary vestibular schwannomas. A flow chart provides an overview of the excluded cases (Fig. 2). The Gardner-Robertson classification was applied when assessing the preoperative pure tone audiograms. This study was performed in line with the principles of the Declaration of Helsinki. Approval was granted by the Clinical Ethics Committee of the authors’ institution (project number: 802/2021BO2).

### Imaging data analysis

**Fig. 3:**
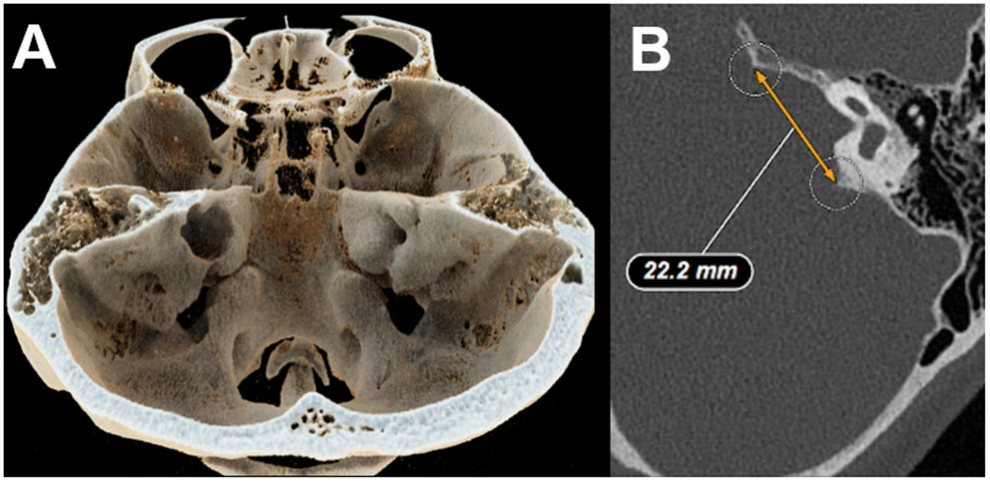
Panel A shows a three-dimensional reconstruction of the widened left IAC and panel B the measurement in the axial preoperative CT-scan

For the calculation of the preoperative tumor size preoperative magnetic resonance imaging (MRI) was used. Cases were excluded if the imaging was older than 6 months. Volumetric size was calculated with the help of the Brainlab® software (Brainlab AG, Feldkirchen, Germany). For the measurement of widening of the IAC at the porus level preoperative computer tomography (CT) was utilized. Axial images were used to measure the widest IAC area. The difference of the tumor side was calculated compared to the healthy side. The parameter was valued as 0 if there was no difference in IAC measurement between both sides or the difference was negative (smaller IAC on tumor side). Figure 3 shows an example of the measurement.

### Immunohistochemistry and immunopositivity scoring

MIB1was used as an established marker for proliferation. Immunohistochemical staining that was done during routine neuropathological workup was reviewed for each case. For quantification of immunopositivity, representative photographs were taken for each tumor, and automated calculation was performed with the Image J software (Version 1.51j8, NIH, Bethesda, 342 MD, USA) and the plugins Bio-Formats (Release 5.4.1; Open Microscopy Environment, 343 Madison, NJ, USA) and ImmunoRatio (Version 1.0c, Institute of Biomedical Technology, University of Tampere, Finland).

### Statistical analysis

The statistical analysis and image preparation were done with the statistic software JMP® version 16.2.0 (Cary, NC: SAS Institute Inc.; 1989). For the uni- and multivariate analyses the continuous variables (age, preoperative tumor volume, IAC widening and MIB1 immunopositivity) were additionally dichotomized to analyze the robustness of the data. For this dichotomization the classification and regression tree (CART) analysis was performed which resulted in specific cut offs for each of the variables. For univariate analyses ANOVA and the Pearson’s Chi-squared test were applied. A linear regression was done to assess IAC widening in a multivariate fashion. Ordinal logistic regression was done for the multivariate analysis of preoperative hearing. A level of significance of α < 0.05 was applied.

## Results

### Patient cohort characteristics

**Fig. 4:**
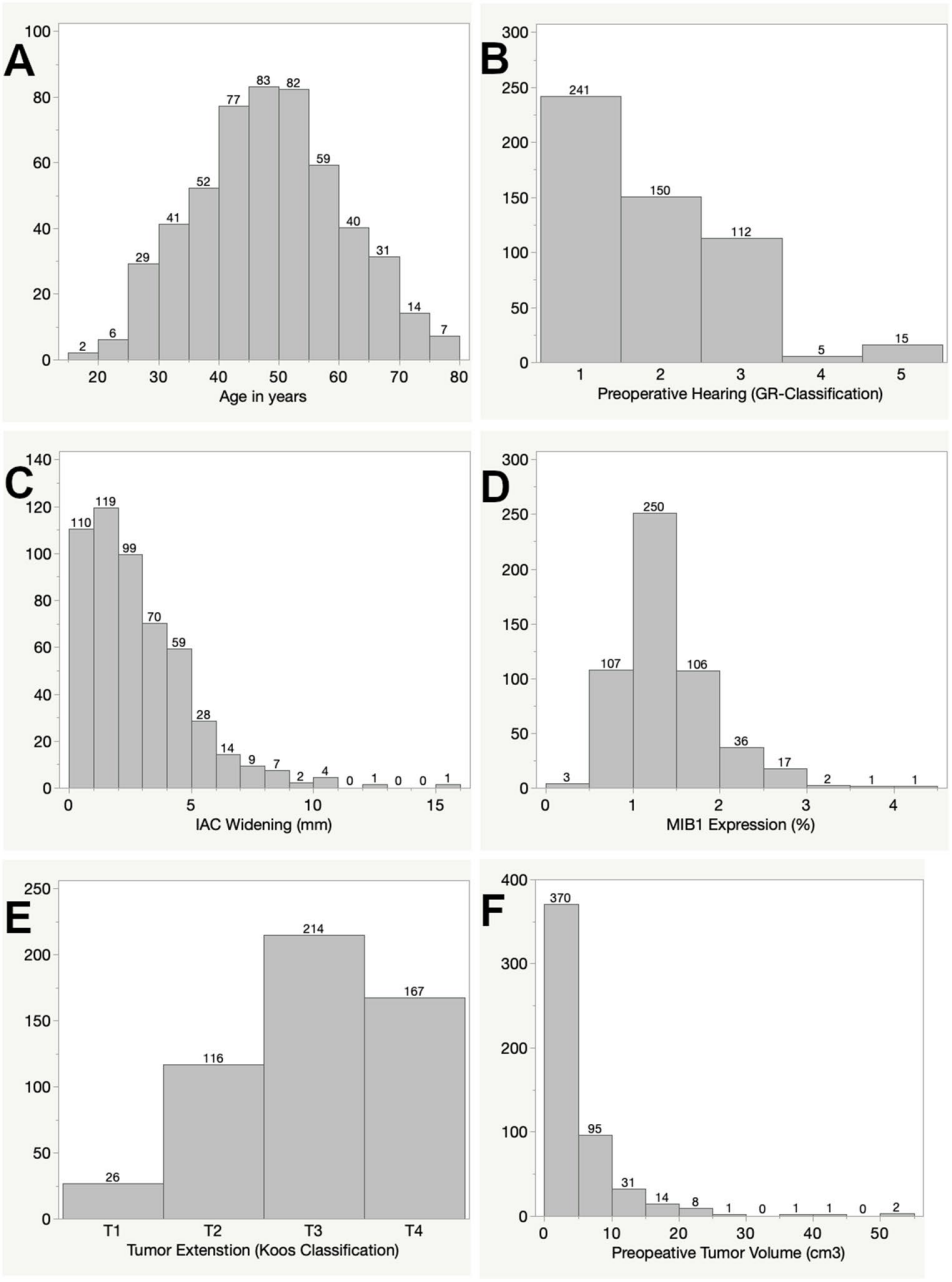
Distribution of clinical factors in the study cohort: age (**A**), preoperative hearing according to the Gardner Robertson classification (**B**), IAC widening in mm compared to the healthy side (**C**), immunohistochemical MIB1 expression in % (**D**), tumor extension according to the Koos classification (**E**) and preoperative tumor volume in cm^3^ (**F**)

The retrospective cohort consisted of 523 primary sporadic vestibular schwannomas from 261 female and 262 male patients, representing a balanced gender ratio. The mean age of the included patients was 48.4 years, ranging from 18.0 to 79.1 years. Preoperative hearing was serviceable in the majority of cases (74.8%). Preoperative pure tone audiograms met the criteria for Gardner Robertson (GR) class 1 in 241 cases (46.1%) and for GR class 2 in 150 cases (28.7%). Non-serviceable hearing was present preoperatively as GR class 3 in 112 cases (21.4%), while GR class 4 and 5 were observed in 5 and 15 patients, respectively (1.0 and 2.9%). Regarding tumor extension according to the Koos classification, most tumors were T3 (*n* = 214, 40.9%) or T4 (*n* = 167, 31.9%), followed by T2 (*n* = 116, 22.2%) and a limited number of purely intrameatal tumors (T1: *n* = 26, 5.0%). The mean preoperative tumor volume was 4.26 cm^3^, with a range from 0.04 to 52.14 cm^3^. Widening of the internal acoustic meatus compared to the contralateral healthy side stretched from non-widened IACs to a difference of 15.1 mm. The mean immunohistochemical expression of MIB1 was 1.34% with a minimum of 0.25% and a maximum of 4.2%. The distribution of these parameters is delineated in Fig. 4; Table 1.

**Table 1:** Distribution of preoperative hearing according to the Gardner-Robertson Classification

	Complete cohort	Preoperative Hearing (Gardner-Robertson Classification)					p-value (Prob > ChiSq)
	n (%)	GR 1	GR 2	GR 3	GR 4	GR 5	
Complete cohort	523 (100)	241 (46.1)	150 (28.7)	112 (21.4)	5 (1.0)	15 (2.9)	-
Gender							
Female	261 (50.0)	117 (44.8)	86 (33.0)	48 (18.4)	3 (1.2)	7 (2.7)	0.2006
Male	262 (50.1)	124 (47.3)	64 (24.4)	64 (18.4)	2 (0.8)	8 (3.1)	
Mean age (in years)	48.4	45.1	49.4	52.0	62.7	58.3	< 0.0001*
Age cut off (in years)							
< 55.47	379 (72.5)	213 (56.2)	99 (26.1)	58 (15.3)	2 (0.5)	7 (1.9)	< 0.0001*
>/= 55.47	144 (27.5)	28 (19.4)	51 (35.4)	54 (37.5)	3 (2.1)	8 (5.6)	
Tumor extension (Koos)							
T1	26 (5.0)	21 (80.8)	1 (3.9)	3 (11.5)	1 (3.9)	0 (-)	0.0055*
T2	116 (22.2)	60 (51.7)	27 (23.3)	25 (21.6)	0 (-)	4 (3.5)	
T3	214 (40.9)	96 (44.9)	71 (33.2)	41 (19.2)	1 (0.5)	5 (2.3)	
T4	167 (31.9)	64 (38.3)	51 (30.5)	43 (25.8)	3 (1.8)	6 (3.6)	
Mean tumor volume (in cm^3^)	4.26	3.38	4.20	5.62	7.32	7.89	0.0013*
Tumor volume cut off (in cm^3^)							
< 6.57	409 (78.2)	203 (49.6)	120 (29.3)	75 (18.3)	3 (0.73)	8 (1.96)	0.0005*
>/= 6.57	114 (21.8)	38 (33.3)	30 (26.3)	37 (32.5)	2 (1.75)	7 (6.14)	
Mean IAC Widening (in mm)	2.69	2.46	2.63	3.18	3.89	2.98	0.0353*
IAC Widening cut off (in mm)							
< 1.2	134 (25.6)	74 (55.2)	41 (30.6)	15 (11.2)	2 (1.5)	2 (1.5)	0.0072*
>/= 1.2	389 (74.4)	167 (42.9)	109 (28.0)	97 (24.9)	3 (0.8)	13 (3.3)	
Mean MIB1 immunopositivity (in %)	1.34	1.33	1.33	1.38	1.30	1.38	0.9049
MIB1 cut off (in %)							
< 1.2	224 (42.8)	109 (48.7)	65 (29.0)	42 (18.8)	2 (0.9)	6 (2.7)	0.7456
>/= 1.2	229 (57.2)	132 (44.2)	85 (28.4)	70 (23 4)	3 (1.0)	9 (3.0)	

Asterisks (*) mark statistically significant differences

### Univariate analysis of factors associated with preoperative hearing

CART-specific cut offs based on the most pronounced differentiation between serviceable (GR 1 or 2) and non-serviceable hearing (GR 3, 4 or 5) were calculated. A higher rate for non-serviceable hearing was observed for age >/=55.47 years, preoperative tumor volume >/= 6.57 cm^3^, IAC widening >/= 1.2 mm and MIB1 immunopositivity >/= 1.2%.

There was a significantly more advanced age of patients with worse preoperative hearing. The mean age increased with each GR class of preoperative hearing (*p* < 0.0001). When dividing the cohort based on the CART-specified cut off at 55.47 years, patients in the younger group had significantly more often serviceable preoperative hearing (82.3%, 312/379) compared to older patients (54.9%, 79/144, *p* < 0.0001).

Regarding tumor extension in the cerebellopontine angle (Koos classification), serviceable preoperative hearing was significantly more common in smaller schwannomas (*p* = 0.0055). T1 tumors showed preoperative serviceable hearing in 84.6% (22/26), T2 in 75% (87/116), T3 in 78.0% (167/214) and T4 in 68.9% (115/167). A more precise look at tumor size revealed that the mean preoperative tumor volume within each GR class was higher with worse preoperative hearing class, ranging from 3.38 cm^3^ for GR 1 to 7.89 cm^3^ for GR 5 (*p* = 0.0013). The CART-specific cut off at 6.57 cm^3^ divided the study cohort into 409 smaller (78.2%) and 114 larger tumors (21.8%), with a significantly lower rate of cases with preoperative serviceable hearing for patients with larger tumors (59.6% vs. 79.0%, *p* = 0.0005).

Widening of the internal acoustic canal also showed a significant association with poor preoperative hearing. The mean IAC widening increased with each higher GR class, except GR 5 (*p* = 0.0353). When dividing the cohort at the CART-specific cut off at 1.2 mm IAC widening, the most pronounced difference in preoperative hearing was described. Cases with less or no IAC widening (*n* = 134) had a serviceable hearing in 85.8% (115/134) compared to tumors with IAC widening of 1.2 mm or more (68.6%, 276 /389).

The mean immunohistochemical expression of the proliferation marker MIB1 was 1.34% throughout the complete cohort, without a significant difference between different GR classes (*p* = 0.9.49) or when using the CART-specific cut off of 1.2% (0.7456).

No difference in preoperative hearing was observed between female and male gender (*p* = 0.2006). The results of the univariate analysis are listed in Table 1.

### Multiple linear regression of IAC widening

Potential factors that were associated with IAC widening were included into a multiple linear regression model. Overall, the regression model was statistically significant (*p* < 0.0001). Younger age (*p* = 0.0049), larger tumor volume (*p* < 0.0001), non-serviceable hearing (*p* = 0.0060) and male gender (*p* = 0.0458) were all independently associated with widening of the IAC. MIB1 expression did not have an independent impact on IAC widening (see Table 2).

**Table 2:** Linear logistic regression for IAC widening

	Estimate	95% Confidence Interval	p-value (Prob > ChiSq)
Intercept	3.97	2.99–4.95	< 0.0001*
Age (in years)	-0.02	-0.04 – <-0.01	0.0049*
Gender (female)	-0.18	-0.36 – <-0.01	0.0458*
Tumor volume (in cm^3^)	0.08	0.05–0.11	< 0.0001*
MIB1 (in %)	-0.03	-0.65–0.049	0.0921
GR1/2 (serviceable)	-0.30	-0.52 – -0.09	0.0060*

Asterisks (*) mark statistically significant results

### Multiple nominal regression for preoperative hearing

When including all relevant parameters that influence preoperative hearing in the univariate analysis into a multivariate model, several factors were identified as independently influencing preoperative hearing. In the multiple nominal logistic regression focusing on serviceable vs. non-serviceable hearing, older age and larger preoperative tumor volume were the parameters with the most pronounced effect, with an odds ratio of 27.60 (CI 9.17–87.18, *p* < 0.0001) and 20.20 (CI 3.43–128.58, *p* = 0.0011), respectively (per change in regressor over the entire range). Additionally, widening of the internal acoustic canal was also an independent significant factor (odds ratio 7.86 (CI 1.77–35.46, *p* = 0.0068). Gender or differences in MIB1 immunopositivity were without statistical significance, but both parameters showed a mentionable statistical trend, with female patients showing a potentially lower odds ratio for poor preoperative hearing (0.69 (CI 0.45–1.06), *p* = 0.0907) and higher MIB1 expression a rather negative trend on preoperative hearing (4.29 (CI 0.87–21.07), *p* = 0.0715).

An additional multiple nominal logistic regression was done with all continuous parameters after CART-specific cut offs based on the best dichotomization regarding serviceable vs. non-serviceable hearing. The analysis rendered similar results. Older age, larger preoperative tumor volume and IAC widening remained independent factors for non-serviceable preoperative hearing (*p* < 0.0001, *p* = 0.0006 and *p* = 0.0044, respectively). A statistical trend for a potentially negative impact of higher MIB1 expression and male gender was also present (*p* = 0.0799 and 0.0795, respectively). Details of the multiple nominal logistic regression analyses are displayed in Tables 3 and 4.

**Table 3:** Multiple nominal logistic regression for serviceable vs. non-serviceable preoperative hearing (according to the Gardner-Robertson classification)

	Estimate (95% CI)	OR (95% CI)	p-value (Prob > ChiSq)
Intercept	-4.96 (-6.25 - -3.74)		< 0.0001*
Age (years)	0.05 (0.04–0.07)	27.60 (9.17–87.18)	< 0.0001*
Preoperative tumor volume (cm^3^)	0.06 (0.02–0.09)	20.20 (3.43–128.58)	0.0011*
IAC widening (mm)	0.14 (0.04–0.24)	7.86 (1.77–35.46)	0.0068*
MIB1 (in %)	0.37 (-0.03–0.77)	4.29 (0.87–21.07)	0.0715
Gender (female)	-0.18 (-0.40–0.03)	0.69 (0.45–1.06)	0.0907

Asterisks (*) mark statistically significant results

**Table 4:** Multiple nominal logistic for serviceable vs. non-serviceable preoperative hearing (according to the Gardner-Robertson classification) with CART-specific parameter cut offs

	Estimate (95% CI)	OR (95% CI)	p-value (Prob > ChiSq)
Intercept	-0.93 (-1.24 – -0.62)		< 0.0001*
Age < 55.47 years	-0.71 (-0.94 – -0.49)	0.24 (0.15–0.37)	< 0.0001*
Preoperative tumor volume < 6.57 cm^3^	-0.41 (-0.65 – -0.18)	0.44 (0.27–0.70)	0.0006*
IAC widening < 1.2 mm	-0.41 (-0.69 – -0.13)	0.44 (0.25–0.77)	0.0044*
MIB1 < 1.2%	-0.20 (-0.41–0.02)	0.68 (0.44–1.05)	0.0799
Gender (female)	-0.19 (-0.41–0.02)	0.68 (0.44–1.05)	0.0795

Asterisks (*) mark statistically significant results

## Discussion

We analyzed the role of IAC widening as a significant predictor for poor preoperative hearing in 523 primary sporadic vestibular schwannomas. In 2001 Tusnoda et al. measured the IAC in 23 VS patients, by fitting an ellipse to measure the length of the axes the direction of the long axis. They observed a larger IAC of the tumor side in all patients. However, there was no correlation with cochlear and vestibular damage and the authors concluded that not only pressure could induced functional damage, but also vascular insufficiency [17]. Although the authors have given new insights into IAC widening in VS, the study is clearly underpowered for allowing conclusions regarding functional hearing impairment.

A few years earlier, in 1997, Matthies et al. assessed changes of the petrous bone as displayed in preoperative imaging and examined potential associations with preoperative hearing in 202 VS. A significant association of IAC diameter difference compared to the healthy side and the probability of preoperative deafness was demonstrated. Furthermore, a higher risk of hearing loss after microsurgical resection was observed, when widening of the IAC was present, suggesting a higher vulnerability of the damaged nerve [18]. Although the authors’ message seems to be quite clear, hearing has only been divided into deafness and residual or normal hearing without a more nuanced approach such as the Gardner-Robertson classification, which was introduced in 1988 [19]. In addition, there are other known factors that influence preoperative hearing function such as patient age and tumor extension. These factors were not included in a multivariate analysis to comprehensively challenge the statistical significance of IAC widening. Our data now confirm that IAC widening is prognostic of preoperative sensorineural hearing, independently of tumor size and patient age, which were both strong predictors by themselves as well. In our cohort the effect remains significant when hearing function is divided into serviceable and non-serviceable hearing, compared to applying the complete Gardner-Robertson scale.

At the time, Matthies and colleagues raised interesting points in their discussion of the pathomechanism of IAC widening and hearing function. They hypothesized that IAC widening may be of benefit for nerve function by reflecting more space for the neurovascular structures of the IAC. However, their data proved otherwise and they concluded that bony widening most likely expressed destructive impact of the tumor on the IAC and that this probably transfers to the nerve as well, leading to hearing impairment [18].

Badie et al. first pointed out a possible connection between heightened intrameatal pressure and the extent of VS in the IAC as well as poor hearing function in 15 VS. They demonstrated a possible association between radiographically assessed IAC tumor area and volume and elevated IAC pressure. Moreover, preoperative hearing function and heightened IAC pressure of 13 VS exhibited no statistical significance but showed a trend in relation to poor hearing function and elevated IAC pressure [15]. Thereupon, Lapsiwala et al. performed similar investigations on a larger cohort of 40 patients with primary VS at the same institution including the 15 patients mentioned [16]. In both studies, hearing loss was classified according to the American Association of Otolaryngology-Head and Neck Surgery (AAOHNS) criteria considering pure tone average and the speech discrimination score [15, 16, 20]. The small number of cases with hearing function data displays a clear limitation of the study from Badie and colleagues [9]. Lapsiwala et al. enlarged the cohort size and included auditory evoked potentials (AEPs) for a subgroup of 20 patients. The authors demonstrated that IAC pressure, that was measured during at the beginning of microsurgical resection by inserting a pressure microsensor, correlated with latency of wave V, expressing preoperative sensorineural hearing impairment [16].

It is obvious that IAC widening may be considered merely as an expression of tumor size, but clinical experience as demonstrated in Fig. 1 shows that tumor size does not necessarily correlate with IAC widening. It seems to be rather a question of how the schwannoma grew in the IAC affecting the bone and neurovascular structures with subsequent pressure. This is further supported by the data, showing that tumor proliferative activity did not contribute to hearing impairment.

IAC widening may therefore be an independent factor predicting preoperative hearing function. To clarify this, IAC widening needs to be regarded in a broader perspective including other factors that are influential on hearing function, such as age, tumor size and growth dynamics. This current study provides these parameters in an exceptionally large cohort and delivers a clear statement. IAC widening is a marker for poor preoperative sensorineural hearing impairment, independent of tumor size and patient age.

### Clinical implications

Since microsurgical techniques, approaches and intraoperative monitoring further evolve, therapy regimes and outcomes will change. Facial nerve and hearing ability preservation have become more important in recent decades. A more precise understanding of hearing-impairing mechanisms in VS might be advantageous in preserving hearing ability and improving therapeutic strategies.

IAC widening stood out as an independent factor associated with poor preoperative hearing ability. Intracanalicular growth [15, 16] as well as IAC widening are connected to impaired hearing, hence both markers might be an argument for resection preserving hearing function and preventing further hearing loss. Furthermore, tumor size exhibited as an independent factor associated with both IAC widening and non-serviceable preoperative hearing. From this it can be concluded that early resection in VS with progressive IAC widening, mainly intracanalicular located VS and large VS, might be beneficial concerning hearing preservation. However, only retrospective preoperative data has been analyzed and we do not yet know whether IAC widening predicts postoperative hearing disability, which should be a matter to focus on in future studies.

### Limitations of the study

The main limitation of the study is its retrospective design and the unavoidable selection bias since it is a purely surgical cohort of a single high-volume center. Naturally, this cohort comprises mainly larger schwannomas. However, the cohort is of large sample size and includes a relevant number of smaller tumors. Additionally, a comprehensive multivariate analysis was done that included all relevant factors and produced a clear result. Nonetheless, it would be necessary to verify our findings in other cohorts of similar sample size and data quality.

## Conclusion

IAC widening is associated with poor preoperative sensorineural hearing, independent of age and tumor size, suggesting pressure induced changes to the bone and subsequent damage to neurovascular structures as pathological mechanism.

## Data Availability

No datasets were generated or analysed during the current study.
